# Outcomes of spontaneous subarachnoid hemorrhage (sah) in neurocritical care unit: a multicenter study

**DOI:** 10.1186/2197-425X-3-S1-A773

**Published:** 2015-10-01

**Authors:** D Iglesias Posadilla, M Gero Escapa, J González Robledo, A Domínguez Berrot, A González Salamanca, L Nogales Martín, M Montero Baladía, A Diego Calvo, M Riesco Crespo, AM Olmos Linares, A Bueno Sacristán, R Ranedo Zaldo

**Affiliations:** Hospital Universitario de Burgos, Burgos, Spain; Complejo Asistencial Universitario de Salamanca, Salamanca, Spain; Complejo Asistencial Universitario de León, León, Spain; Hospital Universitario Río Hortega, Valladolid, Spain; Hospital Clínico Universitario de Valladolid, Valladolid, Spain

## Introduction

SAH is acute cerebrovascular event associated high mortality and tendency to cause dependence, accompanied by multiple medical complications. These patients must be admitted to an intensive care unit and be cared for by a multidisciplinary experienced team.

## Objectives

Analysis of epidemiology, complications and critical care management of SAH patients admitted in five neurocritical care units.

## Methods

Multicenter, prospective and observational study. Including SAH admissions in ICU over 2014. Variables analized: epidemiological, cause of SAH, if aneurysmal SAH: aneurysm location and size, repair treatment; complications, ICU and hospital lenght of stay and morbidity (GOS scale).

## Results

Sample size: 127 patients. Epidemiology data: age 60,46 years (SD 12, 07), 54,33% women and risk factors: HBP 40,16%, dyslipidemia 22,05% and DM 6,30%. Severity scales: Hunt-Hess V: 23,62%, IV 13,39%; Fisher IV: 65,87 %, III 16,67 %; WFNS V: 22,83 %, IV 18,90%. Cause of SAH: 70,97% aneurysmal, 4,03% arteriovenous malformation (AVM) and 25% other. Aneurysm location: anterior comunicant 27,27%; posterior 21,21 %; middle cerebral artery 26,26 %. Aneurysmal sack diameter: small (< 15 mm) 67,05 %, large 22,73% and giant (>25 mm) 10,23%. Repair treatment: surgical 20,63%, endovascular (EVT) 39,68 % and conservative 39,68 %. Time admission-repairment: 3,5 days (SD 11,35), median 1 day (IQR 1). Complications: vasospasm 20,47 %, rebleeding 12,7%, delayed cerebral ischemia (DCI) 26,19%, hydrocephalus 31,75 %, seizures 7,14 %, ventriculitis 6,35% (22,86% with ventricular drainage), heart complications 15,87% and sodium disorders 20,47% (cerebral salt wasting 7,14%, SIADH 2,38% and diabetes insipidus 11,11%). Invasive monitoring: ICP 22,40% and PtiO2 6,60%. Median of length of stay: ICU 5 days (IQR 14) and hospital 15,5 days (IQR 22). Morbidity-GOS scale: 1 (death)= 23,39 % (51,72% donors); 2 = 3,23 %; 3 = 8,06 %; 4: 12,90%; 5: 52,42%.

## Conclusions

The most common cause of SAH is cerebral aneurysm rupture with high Fisher. In this study the endovascular and conservative treatment are the same frequency greater than surgical. Maybe the severity of clinical presentation and high variability in the election of treatment among centers could influence. Time admission-repairment was near to recommendations. Results about complications and GOS scale are similar to the literature.
